# Review: How do spontaneous and sensory-evoked activities interact?

**DOI:** 10.1117/1.NPh.4.3.031221

**Published:** 2017-06-13

**Authors:** Isabelle Ferezou, Thomas Deneux

**Affiliations:** Unité de Neurosciences, Information et Complexité, Centre National de la Recherche Scientifique, Gif-sur-Yvette, France

**Keywords:** spontaneous activity, sensory processing, cerebral cortex, brain states

## Abstract

Twenty years ago, the seminal work of Grinvald et al. revolutionized the view cast on spontaneous cortical activity by showing how, instead of being a mere measure of noise, it profoundly impacts cortical responses to a sensory input and therefore could play a role in sensory processing. This paved the way for a number of studies on the interactions between spontaneous and sensory-evoked activities. Spontaneous activity has subsequently been found to be highly structured and to participate in high cognitive functions, such as influencing conscious perception in humans. However, its functional role remains poorly understood, and only a few speculations exist, from the maintenance of the cortical network to the internal representation of an *a priori* knowledge of the environment. Furthermore, elucidation of this functional role could stem from studying the opposite relationship between spontaneous and sensory-evoked activities, namely, how a sensory input influences subsequent internal activities. Indeed, this question has remained largely unexplored, but a recent study by the Grinvald laboratory shows that a brief sensory input largely dampens spontaneous rhythms, suggesting a more sophisticated view where some spontaneous rhythms might relate to sensory processing and some others not.

## 1 Spontaneous Activity Influences Evoked Responses

Investigating the features detected by individual neurons or by neuronal assemblies has been one of the most successful approaches to understanding brain organization and function. This approach requires measuring the neuronal responses to a set of different sensory inputs; as a consequence, the variability of these responses between different presentations of the same stimulus has long been considered a disturbance that needed to be overcome by trigger-averaging over a number of presentations. This unfortunately led to the disregard of this variability in evoked responses—as well as the large activity fluctuations observed in the absence of stimulation—and rather consider them as noise.

**Fig. 1:**
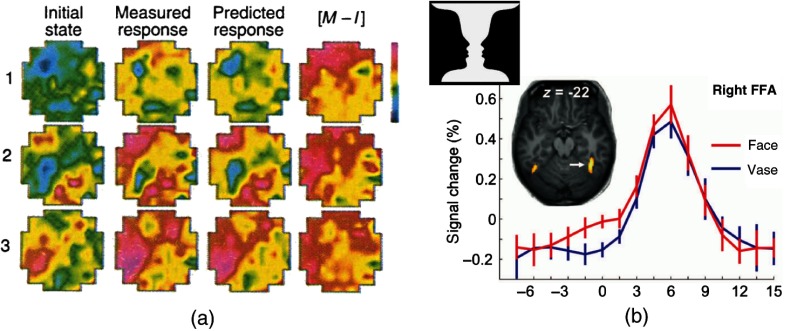
Spontaneous activity influences evoked responses: (a) three consecutive single-trial responses (rows) to the same visual stimulus, showing the initial state, the measured response 28 ms later, and the predicted response obtained by simple summation of initial state and the average response to all trials. Subtracting the initial state from the measured response yielded the net pattern (M−I). Reproduced from Ref. 2 with permission. (b) Peristimulus fMRI signal time courses from right FFA in response to an ambiguous face–vase image (inset), averaged across subjects, after sorting trials according to whether they reported a face or vase perception: face perception was associated with a higher prestimulus activation level. Adapted from Ref. 3 with permission.

Twenty years ago, however, Arieli et al.^1^ focused their interest on these response variabilities and spontaneous ongoing activity, using single-neuron electrical recordings coupled with voltage-sensitive dyes (VSDs) to measure coherent activities in the visual cortex of anesthetized cats. They observed, in particular, that ongoing fluctuations and response variabilities had amplitudes as large as the evoked responses, were highly correlated between neurons as far as 6-mm apart, and showed structure in both space and time. This led them to emphasize the importance of studying these activities as they speculated that the “ongoing electrical activity and its specific interactions with the activity evoked by the stimulus may be one neuronal expression of context.” This speculation was greatly confirmed by their next report^2^ where they showed that the variability in response patterns evoked by individual stimulus presentations could be well accounted for by the ongoing patterns that immediately preceded the stimulation [Fig. 1(a)]. This evidence for integration of a deterministic response to the sensory input with the ongoing network dynamics reinforced their argument that ongoing activity “may provide the neural substrate for the dependence of sensory information processing on context and on behavioral and conscious states.”

They succeeded in triggering a new consideration for ongoing dynamics, and a large number of studies that followed investigated how spontaneous activity patterns influence the responses to specific stimulations.^4^^–^^23^ It is noteworthy that the positive correlation that they reported between ongoing activity and sensory-evoked responses was soon contradicted and that a wider range of interactions was subsequently reported. Indeed, Petersen et al.^7^ observed, in the barrel cortex of anesthetized rats, that sensory-evoked responses were much stronger when ongoing activity was low compared to when it was high. More precisely, the ongoing activity in this preparation showed characteristic up and down states,^24^ where the whole network activity in a local neighborhood alternates between periods of tonic activity (up), possibly propagating as waves, and silence (down). During such synchronized cortical states, which can also be observed during quiet wakefulness,^6^^,^^7^^,^^14^ responses evoked by tactile or tone stimuli are typically of large amplitude and are inversely correlated to the prestimulus membrane potential.^7^^,^^15^^,^^23^ The sensory-evoked cortical responses are further suppressed when the cortex switches from slow wave activity to a more desynchronized state, typical of active wakefulness.^6^^,^^14^ Such suppression of evoked cortical activity occurring during behaviorally active states has been reported both in the primary somatosensory (see also Refs. 25 and 26) and primary auditory cortex.^27^^–^^29^ However, several recent studies indicate that the interaction between behavioral activity, cortical state, and sensory-evoked responses is opposite in the primary visual cortex.^4^^,^^22^^,^^30^^–^^35^ The interplay between ongoing cortical dynamics and sensory inputs, therefore, does not seem to follow common rules across sensory modalities. Furthermore, by recording the membrane potential of mice engaged in a tactile detection task, a recent study from Petersen lab^36^ revealed that, although the ongoing cortical state impacts the evoked sensory response, it has no effect on the performance of the animal.

In the human neuroscience community, the study of ongoing dynamics has met a great interest^3^^,^^37^^–^^42^ (see Ref. 38 for a review). This stems from the interest for high cognitive functions in humans, such as imagination or consciousness, of which ongoing activity could be a hallmark [see also later our mention to the “default mode network (DMN)”]. The influence of ongoing cortical dynamics on the processing of sensory inputs was also established. As an example, Hesselmann et al.^3^ found using fMRI that the perception of a flashed ambiguous face–vase stimulus depended of prestimulation activity level in the fusiform face area (FFA) and an extrastriate visual region specialized for face processing as well as in some other brain areas [Fig. 1(b)]. It thus appears that even conscious perception cannot be considered independently of the “initial state of the system,” to take the author’s words, and that the measured spontaneous activity signals, even though their functional meaning remain unfathomed, constitute at least a fingerprint of this initial state.

## 2 Structure of Spontaneous Activity Reflects Functional Organization and is Influenced by Experience

**Fig. 2:**
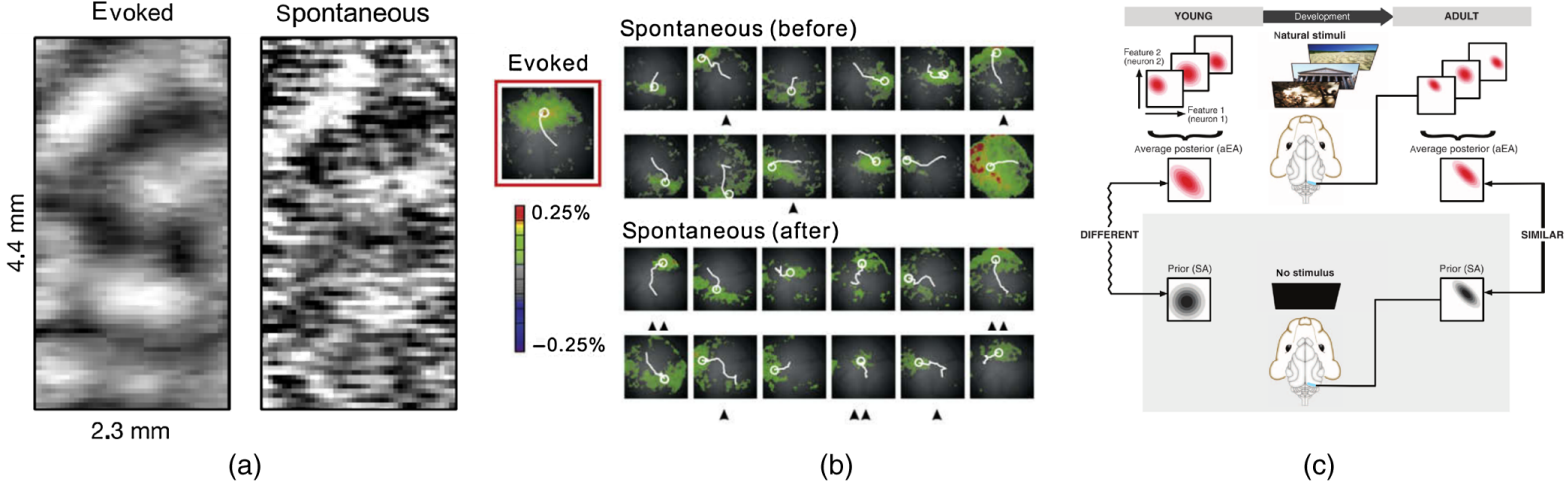
The structure of spontaneous activity reflects functional organization and is influenced by experience: (a) an activation pattern obtained from a single frame from spontaneous activity VSD recording (right) matches the orientation map obtained by averaging responses to full-field gratings of vertical orientation (left), in the visual cortex of an anesthetized cat. Adapted from Ref. 43 with permission. (b) Spontaneous waves recorded using VSD in an anesthetized rat barrel cortex, immediately before and after training with a flashing sequence that evoked the wave template represented on the left. Waves are represented by their first frame and the trajectory of their center over ∼160  ms. Spontaneous waves well matched to the template are indicated by a single arrowhead [correlation coefficient (CC)>0.6] or by double arrowheads (CC>0.7) and are more frequent after training. Adapted from Ref. 45 with permission. (c) Multiunit activity recorded in V1 of awake, freely viewing ferrets either receiving no stimulus (bottom) or viewing natural (top) or artificial stimuli (not shown in this adapted figure) is used to construct neural activity distributions in young and adult animals. Distributions of evoked activities averaged over different stimuli are compared with the distribution of spontaneous activities, assumed to represent the prior expectations about visual features. The internal model of young animals (left) is expected to show little adaptation to the natural environment and thus show a mismatch between spontaneous and evoked distributions. On the contrary, adult animals (right) are expected to be adapted to natural scenes and thus to exhibit a high degree of similarity. Adapted from Ref. 46 with permission.

The Grinvald laboratory made other keystone contributions to the study of spontaneous activity by taking advantage of the exquisite topographical organization of the cat visual cortex, on the one hand, and of VSDs on the other hand, to capture this organization. Functional structures usually revealed by sensory stimulation were also found in the spontaneous dynamics: at the level of a single-neuron functional connectivity,^43^ where population activity maps trigger-averaged on a single-neuron spikes appeared to be near-identical in the resting or stimulation conditions; and at the level of the population representations,^44^ where spontaneous activity patterns were observed, which highly resembled evoked orientation maps [Fig. 2(a)].

That the spontaneous activity reflects the functional organization of the cortical network on which it is riding is not a surprise and has been confirmed at the scale of the whole dorsal surface of cortical hemispheres in mice by VSD imaging.^47^

However, the spectacular aspect of spontaneously emerging orientation maps raised a new question: can the spontaneous cortical states play an active role in sensory processing, as the authors suggested that they might “reflect expectations about the sensory input?”

An additional relationship between sensory-evoked and spontaneous activities lies in the plasticity of the latter, in the sense that sensory-evoked activity can reshape the structure of subsequent spontaneous patterns through learning. This was shown in particular by Dan group^45^^,^^48^ who, after training rats with visual stimuli to evoke wave patterns in their primary visual cortex, observed recalls of these specific patterns in the spontaneous activity during the resting period that followed [Fig. 2(b)]. Such recalls or replays are in fact a phenomenon that is well-known and abundantly studied, in particular, in hippocampal structures.^49^^–^^54^

The similarities in structure between spontaneous and sensory-evoked activities might, therefore, be learned through experience rather than innately. In this light, Berkes et al.^46^ emphasized changes that occur during development, whereby an initial mismatch between the statistics of spontaneous and sensory-evoked (using natural visual stimuli) activities in young ferrets disappears in adult animals [Fig. 2(c)]. There again it is suggested that spontaneous activity reflects prior expectations of “an internal model (of the natural environment) that is adapted gradually during development.”

Moreover, spontaneous activity is known to play an active role during development, in particular, in the early stages of development where propagating waves of activity are known to shape and consolidate the developing networks (see reviews in Refs. 29 and 55, as well as the review from Luhmann^56^ in this issue of *Neurophotonics*). This is a whole field of investigation in itself, and it is not obvious how these spontaneous activities during early development relate to those observed in adults.

Plasticity in the spontaneous activity structure has also been shown in humans. Lewis et al.^57^ found that a stimulated part of the visual cortex modified its resting-state connectivity after training as compared to the untrained part. A few studies have investigated changes in the resting-state network induced by preceding task periods involving memorization or emotional content (see Ref. 38 for review).

Whereas we started this retrospective review with the influence of spontaneous activity on sensory-evoked responses,^2^ we have now discussed influences in the opposite direction through learning. However, another important question has been raised: does the spontaneous activity really embed a representation of “expectations” in such a way that it plays an active role during sensory processing? To address this question, a new level of interaction is envisioned: how are spontaneous dynamics affected by a sensory inflow?

## 3 Sensory Input Switches the Brain Internal Dynamics

### 3.1 Brain Dynamics during a Sensory Input

**Fig. 3:**
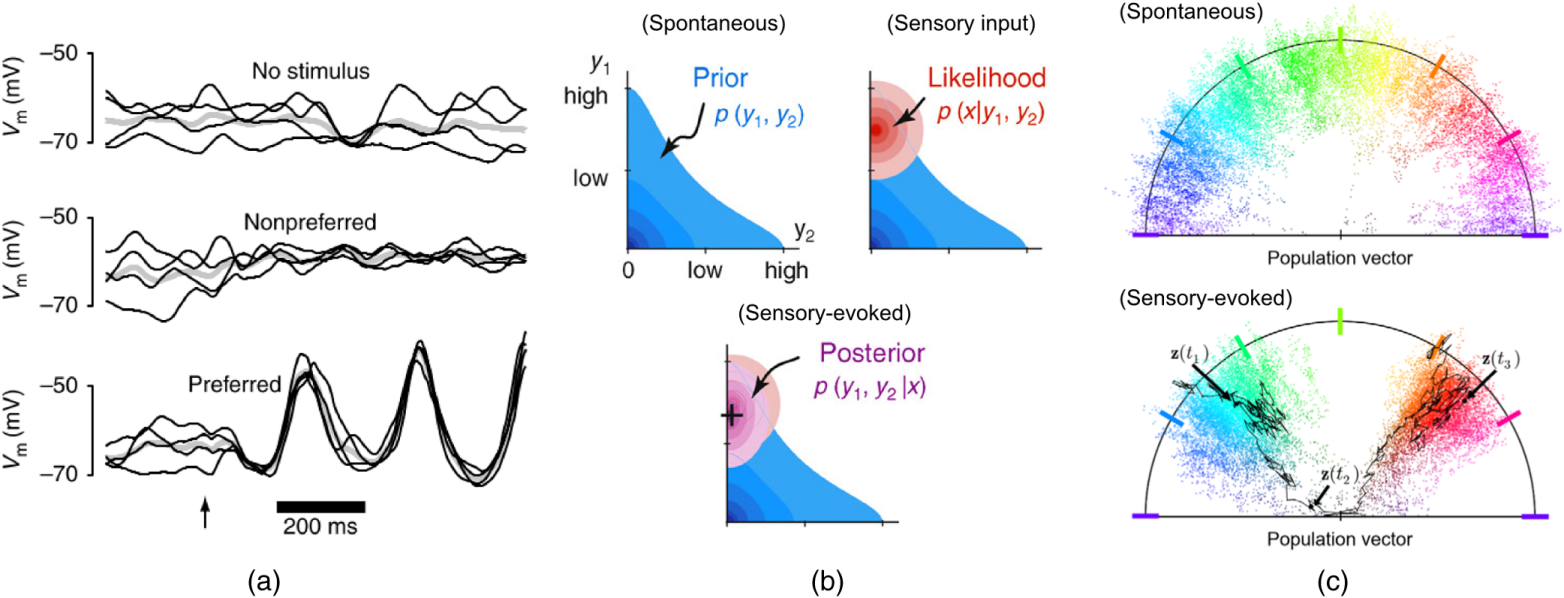
Brain dynamics during a sensory input: (a) example intracellular recordings from a neuron in cat V1. Intertrial variability decreases in stimulated recordings for both nonpreferred (middle) and preferred (bottom) orientations and frequencies of the sine-wave gratings as compared to unstimulated recordings (top). Reproduced from Ref. 58 with permission. (b) Schematic illustration of probabilistic inference in sensory processing. The information brought by *a priori* knowledge about the environment and by the sensory input are represented, respectively, as *a prior* and likelihood probability distributions (top). These two types of information are optimally combined by Bayes’ theorem, forming a posterior probability distribution that displays less uncertainty (is narrower) than the two previous distributions (bottom). Adapted from Ref. 59 with permission. (c) Simulations of a small population of neurons inferring the orientation of a grating by implementing a sampling of the above-mentioned distributions. Individual neurons in these simulations are tuned to different orientations and are preferentially connected to neurons with similar preferences. Individual points in the graphs represent the activity of the full population at different time points, their color and angular position encoding the orientation of the most active neurons, and their radius of the population coherence. In the spontaneous activity, the population activity wanders throughout all orientations, representing the prior distribution (top), whereas, in presence of an ambiguous input, its wandering is restricted to the possible orientations as constrained by the input, representing the posterior distribution (bottom). Adapted from Ref. 60 with permission.

“Stimulus onset quenches neural variability: a widespread cortical phenomenon,”^58^ under this title, a number of well-known neuroscientists gathered 14 different electrophysiology datasets recorded in cats and monkeys, which all showed that intertrial variability decreased in sensory-evoked responses as compared to the preceding period of spontaneous activity [Fig. 3(a)], fluctuations present in the spontaneous activity [Fig. 3(a), top] systematically decreased in amplitude during stimulation, even in instances where this stimulation was not eliciting an “average response” [Fig. 3(a), middle] (Note that, even though it is artificial to split the signals after stimulus onset between an “average response” and “remaining fluctuations,” we chose to call these fluctuations “internal” or “internally generated” activity, as obviously it cannot be called a “spontaneous activity”). This phenomenon actually was already known from intracellular studies^61^ that showed how a sensory input resulted in very reproducible driving of a neuron membrane potential as compared to the spontaneous fluctuations and identified shunting inhibition as a mechanism for the rescaling of the cell excitability.

Even though it appears intuitive that a sensory drive might “clamp” the firing dynamics to fixed patterns and therefore reduce the variability due to random fluctuations generated by the network itself, neural simulations revealed interesting properties of this general effect. For example, the work of Abbott group^62^^,^^63^ showed not only that variability reduction was an intrinsic property of interconnected networks shifting from chaotic to driven dynamics when exposed to an input but also that complex nonlinear interactions occurred between intrinsic and sensory-driven dynamics. These included the preference for some input frequency without any resonance effect, the drive at harmonic frequencies initially not present in the input, and the curving of the spatial patterns of the input toward those of the intrinsic dynamics. Some of these effects were later confirmed experimentally.^64^ On the other hand, other computational neuroscientists have advocated that the reduction of variability corresponds to a very peculiar structural property of the brain network, such that its activity spans a highly multidimensional space “at the edge” of multiple bifurcations, leading to multiple “ghost attractors.”^65^^,^^66^ In the absence of a sensory input, the spontaneous activity can visit a large repertoire of states; however, even a weak external input can lead it to fall into one of the attractors, which decreases variability.

In addition to this modeling effort, a functional role in sensory processing was proposed:^59^^,^^60^^,^^67^ the spontaneous activity, by sampling a large ensemble of states, maintains an internal representation of all possible external environments and thereby implements an “expectation” or, in Bayesian terms, a “prior.” Once combined with the information brought by a sensory input about the actual state of the external world, this prior is reshaped into a “posterior,” which by essence embeds less uncertainty and therefore restricts the number of sampled states [Figs. 3(b) and 3(c)].

According to this theory, the spontaneous activity is viewed as playing an active role in sensory processing, the network being permanently in an attempt to make inferences about the environment, even under resting condition or sleep where it explores all possibilities learned from accumulated experience. Because sensory-evoked activity is then viewed as a combination of the information entailed in the internal dynamics, on the one hand, and in the sensory input, on the other hand, it gives a functional significance to the above-mentioned patterns of integration between spontaneous fluctuations and sensory-evoked activities, as well as the reshaping of spontaneous activity by experience. This view, however, appears quite restrictive in regard to some stereotypic and widespread spontaneous rhythms, which are unlikely to achieve a “sampling of internal representations,” such as the up and down fluctuations.^24^

### 3.2 Brain Dynamics after a Sensory Input

**Fig. 4:**
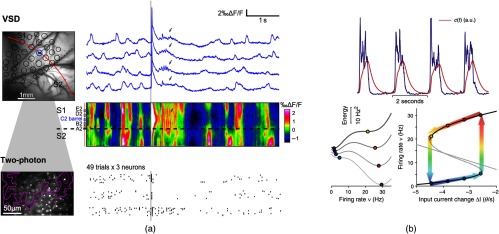
Brain dynamics after a sensory input: (a) spontaneous dynamics, response evoked by a brief somatosensory stimulation and poststimulation dynamics, recorded in anesthetized rat barrel cortex using both VSD imaging of population activity and two-photon microscopy of individual neurons spiking activity. VSD signals display four example trials and are extracted from the stimulated barrel location (blue circle in the barrel map on the left); in addition, a spatiotemporal display allows visualizing activity throughout a line (marked in red in the barrel map) crossing both the primary and secondary somatosensory cortices (S1 and S2). Raster plot of spikes extracted from the two-photon recordings displays 49 trials for three different neurons. The main effect of the sensory input on the subsequent dynamics is a decrease or even interruption of the up states appearances for a few seconds (see text for more details). Adapted from Ref. 68 with permission. (b) A model of up and down states generation that involves a fatigue mechanism. (top) Example multiunit activity trace from experiment (blue) and reconstructed fatigue variable c(t). (bottom left): energy landscapes for different levels of activity-dependent adaptation/fatigue; when fatigue is high (top trace), the minimum energy point is an absence of activity. (bottom right): stable (solid branches) and unstable (dotted branch) asymptotic states of firing rate at different fatigue levels here represented as effective changes in the input current to the neurons in the network; colored arrows and circles depict the orbit in the phase plane followed by the network under the relaxation oscillator regime. Adapted from Ref. 69 with permission.

To further investigate the interactions between spontaneous and evoked activities, Deneux and Grinvald^68^ explored how the internal dynamics would be modified “after” a brief sensory input. In the barrel cortex of anesthetized rats, with a preparation that displays the stereotypical up and down states, the authors observed that even after a brief single whisker stimulation, this rhythm was significantly perturbed for several seconds, with up events failing to occur, in particular, in the stimulated barrel-related column [Fig. 4(a)]. As a result, the interaction between internal (recurrent, top-down) and feedforward activities did not appear any more as the smooth integration of two complementary activities, but on the contrary as a competition between orthogonal activities. The authors indeed suggested that “at the onset of a sensory input, some internal messages are silenced to prevent overloading of the processing of relevant incoming sensory information.” In addition, this switch in the internal dynamics was also characterized by a transient burst of activity at around 15 Hz (identified as a thalamo-cortical oscillation)^70^^,^^71^ and a transient activity increase of a small fraction of the neurons [both visible in Fig. 4(a); the ~15-Hz activity is marked with gray arrows]. These two patterns occur identically as well after the onset of a longer, sustained stimulation (not visible in the figure, see Ref. 68 for details) and thus appear as a stereotyped sequence of events that take place in the presence of a new sensory stream. Finally, the internal activity decrease showed some spatial organization as it was maximal in the stimulated barrel location [visible in Fig. 4(a)], indicating that local mechanisms might be at work.

In this report, emphasis is put on a rupture between pre- and poststimulus onset activities, with the notion that specific switching occurs, affecting subsequent dynamics at the temporal scale of seconds. To this respect, the authors of this review also present in this issue of *Neurophotonics* a research article of particular interest as it reproduces in an awake monkey V4 area the variability “quenching” reported by Churchland et al.^58^ (yet adding the precision that the activity that is suppressed is a specific global and low-frequency fluctuation). It further shows that this suppression already occurs with maximal strength from the lowest contrast, suggesting that a specific switch occurs rather than a continuous integration.

The notion that an input may cause a switch in the dynamics of a network activity is already present in computational models of the brain^65^ and has been reported in *in-vitro* studies^72^^–^^74^ where microstimulations remarkably induced transitions between up and down, or between synchronized and desynchronized states, as well as *in vivo*, using nonphysiological stimuli.^75^ Also, it has been known for a long time in the human neuroscience community that sensory inputs alter synchronized rhythms, with the most famous effect being the decrease in alpha rhythms,^76^^–^^78^ and that resting-state activity in the DMN decreases upon stimulation.^39^^,^^42^^,^^79^

It is in fact expected that a new sensory input might cause major changes in the global brain state, switching it, for example, from quiet to active or from asleep to conscious. Studying the details and mechanisms of these switches in addition to the mechanisms of specific rhythms taken in isolation will probably provide new insights on these complex properties of the brain network. As an example, checking how existing models of generation of the up and down fluctuations^69^^,^^80^^,^^81^ [Fig. 4(b)] would predict not only the evoked responses^18^^,^^82^ but also subsequent internal activity changes is warranted.

### 3.3 Global Network Changes

The changes in cortical dynamics induced by the presence of a sensory input occur also at the scale of the full brain, indicative of a change of the subject global state. This is the topic of functional connectivity studies in humans, for which the imaging techniques (EEG, MEG, fMRI, PET), characterized by coarse spatial resolution but access to the whole brain at once, are particularly adapted. A set of structurally and functionally connected brain regions specifically deactivated during tasks that demand attention to external stimuli and innovative events has been collectively named the “DMN.” Although the understanding of its role in brain function still remains largely elusive, the implication of the DMN in internal modes of cognition (autobiographical memory, self-referential thought, and mind-wandering) as well as its alterations in neuropsychiatric disorders is the subject of intense research efforts.^42^^,^^79^ The ongoing DMN activity has been reported to be negatively correlated with stimulus-induced responses and perception in humans;^83^^,^^84^ however, positive correlation has been also observed,^85^ suggesting that the experimental context and the behavioral paradigm strongly impact the link between DMN activity and sensory processing.

## 4 Not One but Many Spontaneous Activities

Altogether, it appears that, despite considerable efforts aimed at studying the spontaneous activity, its functional role remains elusive and might range from low-level maintenance and consolidation of the network^86^^,^^87^ to high-level signature of consciousness.^39^^,^^42^^,^^79^ In particular, even though it is undisputable that ongoing states interfere with sensory processing and are reshaped by learning, direct experimental testing of whether they take an active role in sensory processing remains difficult.

Obviously, it is a pitfall anyway to consider spontaneous activity as a homogeneous phenomenon, as it entails all neural processes, unconscious and conscious, that are not directly (or at least not easily) accessible to probing by identified stimulations or tasks. Even the apparent same rhythms in different contexts can in fact display important structural differences, as was shown with slow frequency activity that appeared to be more local during slow wave sleep compared to during quiet wakefulness.^88^^,^^89^

However, the impressive development of *in vivo* optical methods, pioneered in particular by Grinvald, which allow probing cortical spatiotemporal dynamics at the single-trial level in both anesthetized and awake preparations, will undoubtedly keep on bringing precious keys to further unravel the functional interplay between internal dynamics and sensory inputs in cortical networks.
